# Multi-Institutional Sharing of Electronic Health Record Data to Assess Childhood Obesity

**DOI:** 10.1371/journal.pone.0066192

**Published:** 2013-06-18

**Authors:** L. Charles Bailey, David E. Milov, Kelly Kelleher, Michael G. Kahn, Mark Del Beccaro, Feliciano Yu, Thomas Richards, Christopher B. Forrest

**Affiliations:** 1 Children’s Hospital of Philadelphia, Philadelphia, Pennsylvania, United States of America; 2 Perelman School of Medicine, University of Pennsylvania, Philadelphia, Pennsylvania, United States of America; 3 Nemours Children’s Hospital, Orlando, Florida, United States of America; 4 Nationwide Children’s Hospital, Columbus, Ohio, United States of America; 5 Children’s Hospital of Colorado, Aurora, Colorado, United States of America; 6 Seattle Children’s Hospital, Seattle, WA;; 7 St. Louis Children’s Hospital, St. Louis, Missouri, United States of America; The University of Auckland, New Zealand

## Abstract

**Objective:**

To evaluate the validity of multi-institutional electronic health record (EHR) data sharing for surveillance and study of childhood obesity.

**Methods:**

We conducted a non-concurrent cohort study of 528,340 children with outpatient visits to six pediatric academic medical centers during 2007–08, with sufficient data in the EHR for body mass index (BMI) assessment. EHR data were compared with data from the 2007–08 National Health and Nutrition Examination Survey (NHANES).

**Results:**

Among children 2–17 years, BMI was evaluable for 1,398,655 visits (56%)**.** The EHR dataset contained over 6,000 BMI measurements per month of age up to 16 years, yielding precise estimates of BMI. In the EHR dataset, 18% of children were obese versus 18% in NHANES, while 35% were obese or overweight versus 34% in NHANES. BMI for an individual was highly reliable over time (intraclass correlation coefficient 0.90 for obese children and 0.97 for all children). Only 14% of visits with measured obesity (BMI ≥95%) had a diagnosis of obesity recorded, and only 20% of children with measured obesity had the diagnosis documented during the study period. Obese children had higher primary care (4.8 versus 4.0 visits, p<0.001) and specialty care (3.7 versus 2.7 visits, p<0.001) utilization than non-obese counterparts, and higher prevalence of diverse co-morbidities. The cohort size in the EHR dataset permitted detection of associations with rare diagnoses. Data sharing did not require investment of extensive institutional resources, yet yielded high data quality.

**Conclusions:**

Multi-institutional EHR data sharing is a promising, feasible, and valid approach for population health surveillance. It provides a valuable complement to more resource-intensive national surveys, particularly for iterative surveillance and quality improvement. Low rates of obesity diagnosis present a significant obstacle to surveillance and quality improvement for care of children with obesity.

## Introduction

Assessing the health and healthcare of the nation’s children depends critically on data that are timely, relevant, and accurate. Currently, surveillance and policy decisions rely heavily on labor-intensive periodic and *ad hoc* surveys using cross-sectional designs [1]. However, electronic health records (EHRs) are rapidly coming into wider use as the medium in which information about care of patients is recorded [2]; in the United States, the ‘Meaningful Use’ initiative for EHR systems [3] [4] is providing a significant stimulus for this process. The shift of health information into electronic forms more amenable to analysis and exchange creates opportunities to improve healthcare quality, develop new methods for clinical research, and follow the health of patient populations using EHR-derived data [5]. Several policy analysts have suggested that multi-institutional sharing of EHR data presents a new paradigm for advancing population health [6] [7] [8]. In particular, greater facility with EHR data is critical to achieving a learning health system [9] [10], in which information derived from clinical care continuously supports advances in medical understanding and delivery of health care. These advances will be of particular value to child health research, much of which relies on longitudinal changes in growth, health, and development.

Realizing the potential of EHR-derived data requires addressing important questions, including differences in representation of information, variability in data capture, and governance issues [11]. Early efforts include the eMERGE Consortium, which used site-specific combinations of discrete data and natural language processing to identify patients with particular diagnoses that could be pooled for genotype-phenotype studies [12]. The HMO Research Network [13] has piloted a system that allows queries against member sites’ records; the Shared Health Research Information Network’s (SHRINE) query framework operates across sites that have implemented compatible infrastructure using i2b2 [14]. These tools provide for addressing aspects of heterogeneity between EHRs to support federated case ascertainment for research, but require significant resources to implement the required infrastructure. In parallel, mechanisms for patient-centered health information exchange supporting clinical operations and continuity of care are rapidly evolving [15], with significant involvement in the United States from the Office of the National Coordinator for Health IT [16]. Processes that benefit from frequent iteration, such as quality improvement and public health surveillance, may benefit from hybrid strategies that incorporate limited start-up cost and well-defined data models.

Groups of institutions having unified EHRs, such as the Kaiser Permanente system [17], the Harvard Vanguard Medical Associates [18], and the Nemours foundation [19] have demonstrated the utility of aggregating data to examine larger populations of patients in pediatric as well as adult health care, and analyses based on data from a single EHR are becoming more common. At the same time, we are better defining potential limitations of EHR-derived data [20] and developing ways to address them [21] [22]. Sharing of data from disparate EHR systems to enable population-based research [9] is a logical and widely anticipated extension. As we evolve toward this goal, it will be important to study not only the mechanisms for data sharing, but also the ability of data aggregated through various means to support valid and meaningful conclusions about population health.

America’s children, like the population as a whole, are experiencing alarming levels of obesity, although rates appear to have stabilized at 17–19% of children and adolescents [23] [18]. National estimates of childhood obesity are generated by the National Health and Nutrition Examination Survey (NHANES) [24]. Data are combined over two-year intervals to accrue sufficient subjects to generate precise estimates. Sharing of EHR-derived anthropometrics can achieve very large sample sizes to generate interval assessments more rapidly, provide multiple assessments per child to permit longitudinal assessment, and link with other relevant clinical data. In these ways, it can serve as a valuable complement to more in-depth but resource-intensive structured population surveys such as NHANES. This sort of population health surveillance is a key goal of the Meaningful Use initiative, and early efforts to examine feasibility are underway [15], but there are few examples to date in pediatrics.

We report here a study testing the feasibility, validity, and utility of multi-institutional EHR data sharing for monitoring and investigating childhood obesity. The study was expressly designed to minimize resources required by contributing institutions, using processes similar to those used for quality improvement, or for the health information exchange envisioned in the Meaningful Use initiative as a part of routine EHR interoperation. Six pediatric academic health systems from different regions of the United States participated, sharing data from 2007–2008, to examine both the logistical requirements for data sharing and the characteristics of EHR-derived data. The interval matches a data-reporting period for NHANES, to better compare the two approaches. To further assess the utility of EHR-derived data, we examined the association of measured obesity with other clinical data, including the diagnosis of obesity, detection of co-morbidities, and assessment of healthcare utilization. Our goal was to explore the unique potential of EHR-derived data to provide an integrated clinical picture over time.

## Methods

### Ethics Statement

The Institutional Review Boards at the Children’s Hospital of Philadelphia, Nemours Children’s Hospital, Nationwide Children’s Hospital, Children’s Hospital of Colorado, Seattle Children’s Hospital, and St. Louis Children’s Hospital approved this study protocol, and granted waivers of individual consent based on absence of individually identifying data. Individual subject identifiers from the EHR were replaced, dates of all visits for a given subject were shifted by a random offset, and subjects’ age at each visit was recorded in months.

### Data Acquisition

The study was conducted in the Pediatric EHR Data Sharing Network (PEDSNet), a consortium formed in late 2009 in response to the Institute of Medicine’s call for development of real-world examples of learning health systems [9] [10]. Each site extracted from their EHR information from all outpatient physician visits in 2007–2008, excluding emergency department and surgical center visits, for all patients with age <18 years. Data included subject sex and age, visit date and department specialty, subject’s measured weight and height, and all diagnoses recorded for the visit. Four of the six institutions use the EpicCare EHR [25], one uses Cerner Millennium [26], and one uses Allscripts [27]. Since the Allscripts EHR does not associate diagnoses with a specific visit, that site reported all diagnoses listed as active on the date of the visit. Data were transmitted to the coordinating center, where analytic databases were constructed and quality control tests were run. Ambiguities or apparent errors were corrected by communication with the submitting site and resubmission. Sites reported the feasibility of capturing requested elements, as well as required resources for regulatory review, query definition, and data extraction and de-identification.

For analyses using the NHANES 2007–2008 samples, DEMO and BMX datasets were retrieved from the NHANES web site [24] and age, sex, weight, stature, and MEC sample weight were used for analyses.

### Determination of Overweight and Obesity

Body mass index (BMI) was calculated in kg/m^2^. Visits for which any of age, sex, or weight were missing were excluded. If measured height was not available for the visit, but values were available for the prior and subsequent visits, height was imputed using linear interpolation. If two or more height values were available only before or after the current visit and yielded consistent percentiles on the NHANES 2000 height-sex-age growth curves, a value for the current visit was imputed based on the corresponding percentile at the current visit. The subject’s age, sex, and BMI were then used to calculate a percentile based on the NHANES 2000 curves, using the SAS algorithm published by the CDC. [28] BMI values flagged as outliers (z-score<−4 or >5) using the CDC’s modified z-score algorithm [29] were excluded, in keeping with accepted norms. A subject was considered obese if the percentile was 95 or greater, and overweight if the percentile was at least 85 but less than 95.

A diagnosis of obesity was noted if any of the ICD-9-CM codes 278, 278.0, 278.00, 278.01, 278.1, 759.81, 783.1, or V85.54 were present. For specialty-specific analyses, only visits in the same specialty were considered for scoring both BMI and diagnoses, though imputation of height was allowed using data from other visit types, as these data would be accessible in the EHR. For person-level analyses, BMI criteria or diagnostic criteria could be met independently at any eligible visit during the study period.

### Detection of Obesity-Related Co-Morbidity

For children with evaluable BMI, recorded diagnoses from all visits were clustered into clinically homogeneous categories using Expanded Diagnostic Clusters (EDCs) from the Adjusted Clinical Group (ACG) System [30]. Within each category, we calculated a standardized morbidity ratio of prevalence in children with obesity versus that in the cohort as a whole. Rates were standardized by age and sex.

### Data Analysis

Data management was done using Perl 5.12–5.16 [31] and MySQL 5.5 [32]. Analyses were done using R 2.14 or 2.15 [33] and SAS 9.2 or 9.3 software [34].

For EHR-derived data, estimates of prevalence were computed as unweighted proportions of the indicated population; for NHANES data, MEC sample weights were used. At the recommendation of NHANES staff, no BMI values were excluded as outliers, in order to better match the methods used in their published analyses. A two-tailed Student’s *t*-test was used to assess significance of continuous variables, and χ^2^ testing was used for categorical variables. Curves of average BMI values by age were fitted in R using cubic polynomial regression. To assess the reliability over time of BMI, per-subject intraclass correlation coefficients (ICC2) were computed [35]. Multiple linear regression incorporating age, sex, obesity, and comorbidity burden as encoded by ACG Relative Utilization Bands [30] was performed using R.

## Results

### Dataset Construction

Sites reported between 5 and 40 person-hours required for retrieval of data elements from their EHR, with the majority of effort being in query construction and regulatory review. Overall, sites required 0–2 revisions to their extraction process, after review by the data coordinating center, to resolve data quality issues. In two cases, sites had systematic errors in their initial data involving calculation of age at visit; of note, both were detectible as outliers in the resulting BMI distributions by comparison to the other sites, without reference to external standards. Age and sex were consistent within subjects across >99% of visits. Anthropometric data were also internally consistent, with <0.35% of visits recording apparent English-metric unit errors based on prior or subsequent visits.

### Population

**Figure 1:**
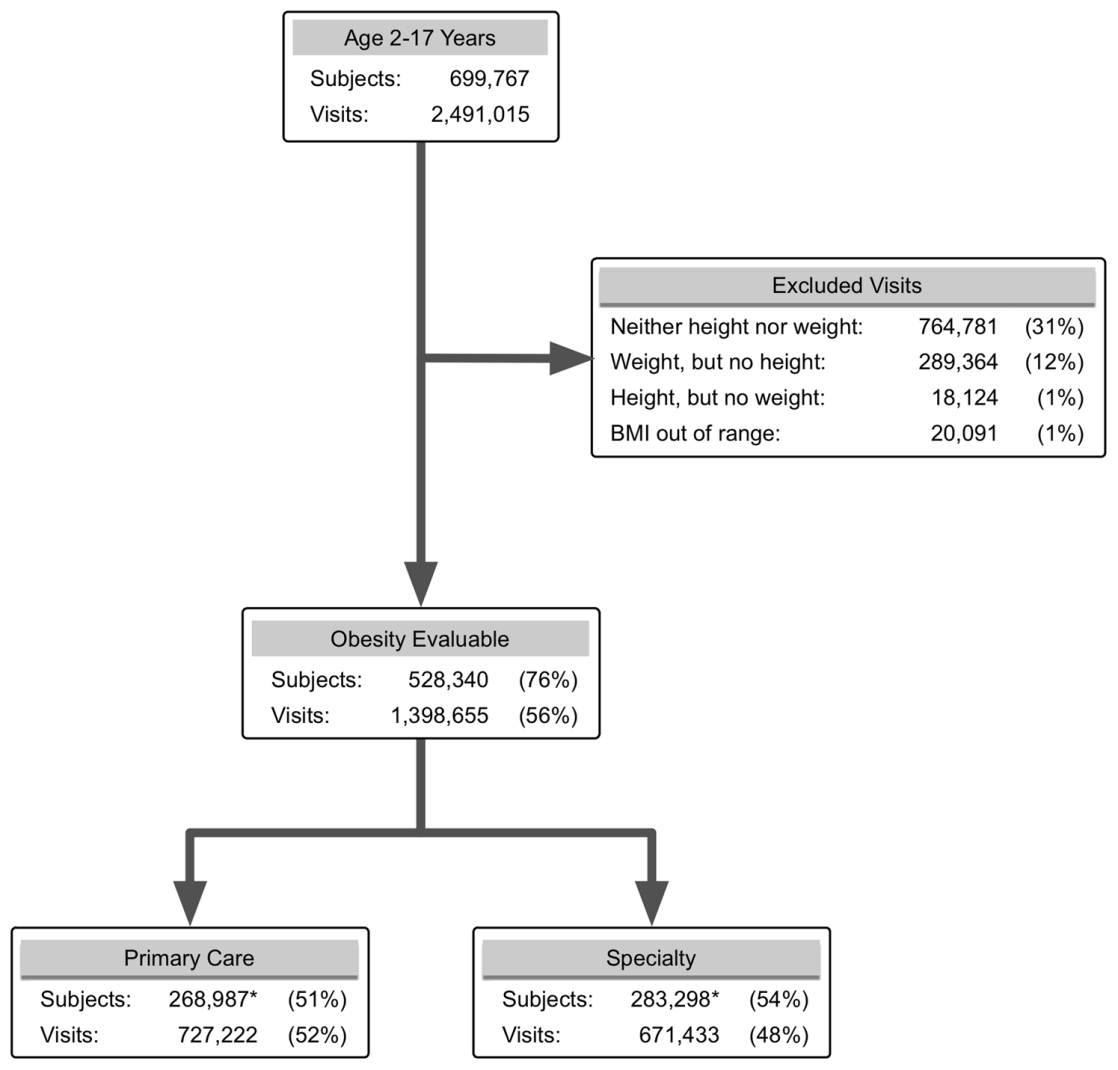
Evaluable Population for Obesity Analyses. Development of the dataset for obesity-related analyses, showing the number of evaluable children and visits at each step. Percentages at each step are calculated relative to totals in the prior step. Since patients may have both primary care and specialty visits, subject counts at this step do not sum to the prior total; these values are marked with an asterisk.

The EHR dataset included 2,491,015 outpatient visits involving 699,767 children 2–17 years of age. Of these, 1,398,655 visits (56%) made by 528,340 children (76%) had sufficient data to compute a BMI (**Figure 1**), with a mean of 2.6 (range 1–141; inter-quartile range 1–3) BMI assessments/child. Height was imputed for 21% of these visits. For every month of age from 2–15 years, the dataset contained over 6,000 BMI measurements., Counts decreased steadily for adolescents ages 16–18, to a low of 1674 observations for children 215 months old, likely representing transition of older adolescents out of pediatric care.

Fifty-two percent of subjects were male. Of total visits, 51% were at primary care sites and 49% at specialty clinics; 28% were made by children 2–4 years of age, 39% 5–10 years of age, and 34% 10–17 years of age. Contributions from a single site ranged from 3 to 35% of subjects and 2 to 43% of visits. All proportions were comparable for evaluable visits.

### Measurement of Obesity and Overweight

**Figure 2:**
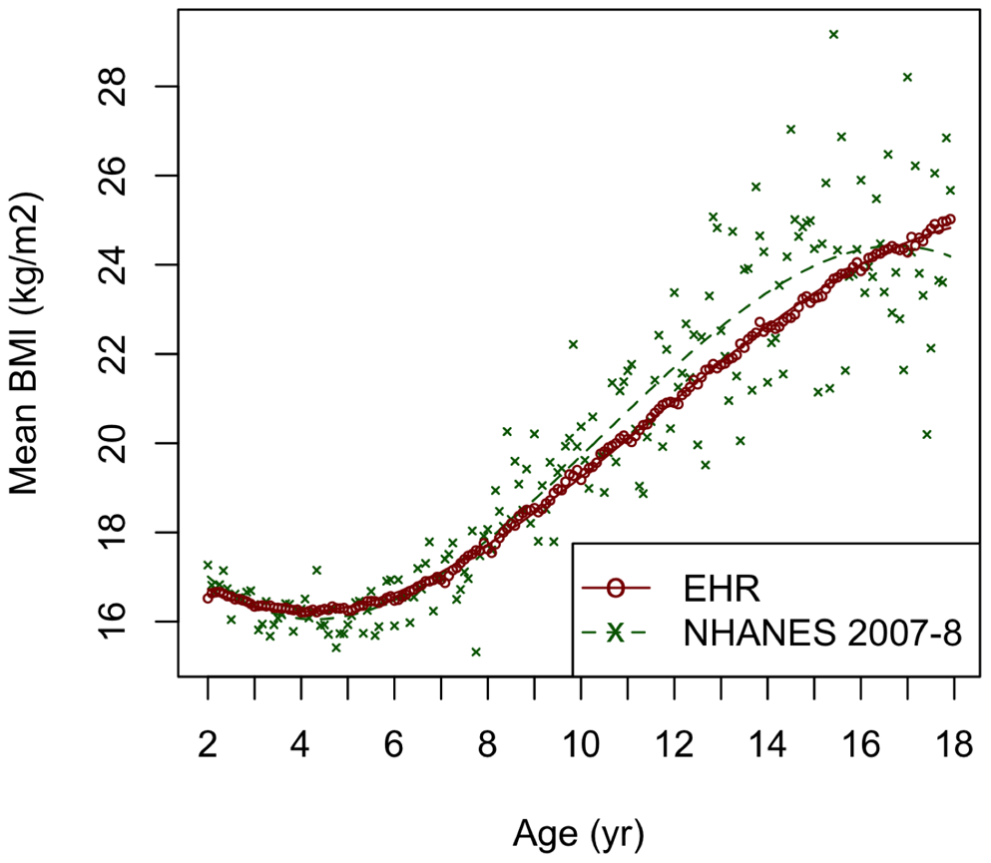
Comparison of EHR and NHANES 2007–8 Cohorts. Average measured BMIs for children of both sexes at each month of age from 2–17 years in the multi-institutional EHR cohort and in the NHANES 2007–8 cohort. In addition to individual points, curves fitted to each dataset by cubic polynomial regression are shown.

**Figure 2** shows a comparison of BMI measurement in the clinical data from the EHR dataset to the U.S. benchmark NHANES survey for the same period. Mean BMI values for each month of age were highly similar in the EHR and NHANES datasets. However, there was substantially higher precision in the EHR-derived data, particularly among adolescents.

BMI measurements were used to estimate the prevalence estimates of obesity and overweight in different age groups, as shown in **Table 1**. The estimates produced using EHR-derived data were 18% for obesity and 35% obesity plus overweight; these figures align closely with the 18% and 34% estimates, respectively, derived from the NHANES surveys. The differences between EHR-based and NHANES estimates were slightly greater for 2–4 year old children, but they did not reach significance.

**Table 1:** Prevalence of Obesity and Overweight in EHR-Derived Data and NHANES Data.

	Fraction of sample^b^	% Obese	% Overweight, never obese
**NHANES 2007–8** a			
*2–17 years*	1.000	18	16
* 2–4 years*	0.194	11	12
* 5–10 years*	0.349	19	15
* 11–17 years*	0.457	20	17
**Multi-site EHR Data**			
*2–17 years*	1.000	18	17
* 2–4 years*	0.280^c^	14	16
* 5–10 years*	0.418^c^	18	17
* 11–17 years*	0.374^c^	20	17

a All proportions for NHANES data were calculated using MEC sample weights; no BMI outliers were excluded in prevalence estimates following NHANES standard practice.

b Total raw samples sizes were 3032 for NHANES and 528,340 for multi-site EHR data.

c Different visits for a given child may appear in different age subgroups, due to the longitudinal nature of the EHR dataset. Therefore, the fractions of children from each age subgroup do not sum to 1.000.

EHR: Electronic Health Record. NHANES: National Health and Nutrition Examination Survey.

Because the dataset contained 101,897 obese or overweight children with multiple visits, we were able to assess the stability over time of EHR-based BMI measurements by calculating the per-child intraclass correlation coefficient (ICC). For obese children, the ICC was 0.90, for overweight but non-obese children 0.81, and for all children 0.97, demonstrating that clinical BMI assessment was a highly reliable process. Among children who were obese at any visit, 85% remained obese or overweight at all visits during the study period.

### Correlation with Clinical Practice

**Figure 3:**
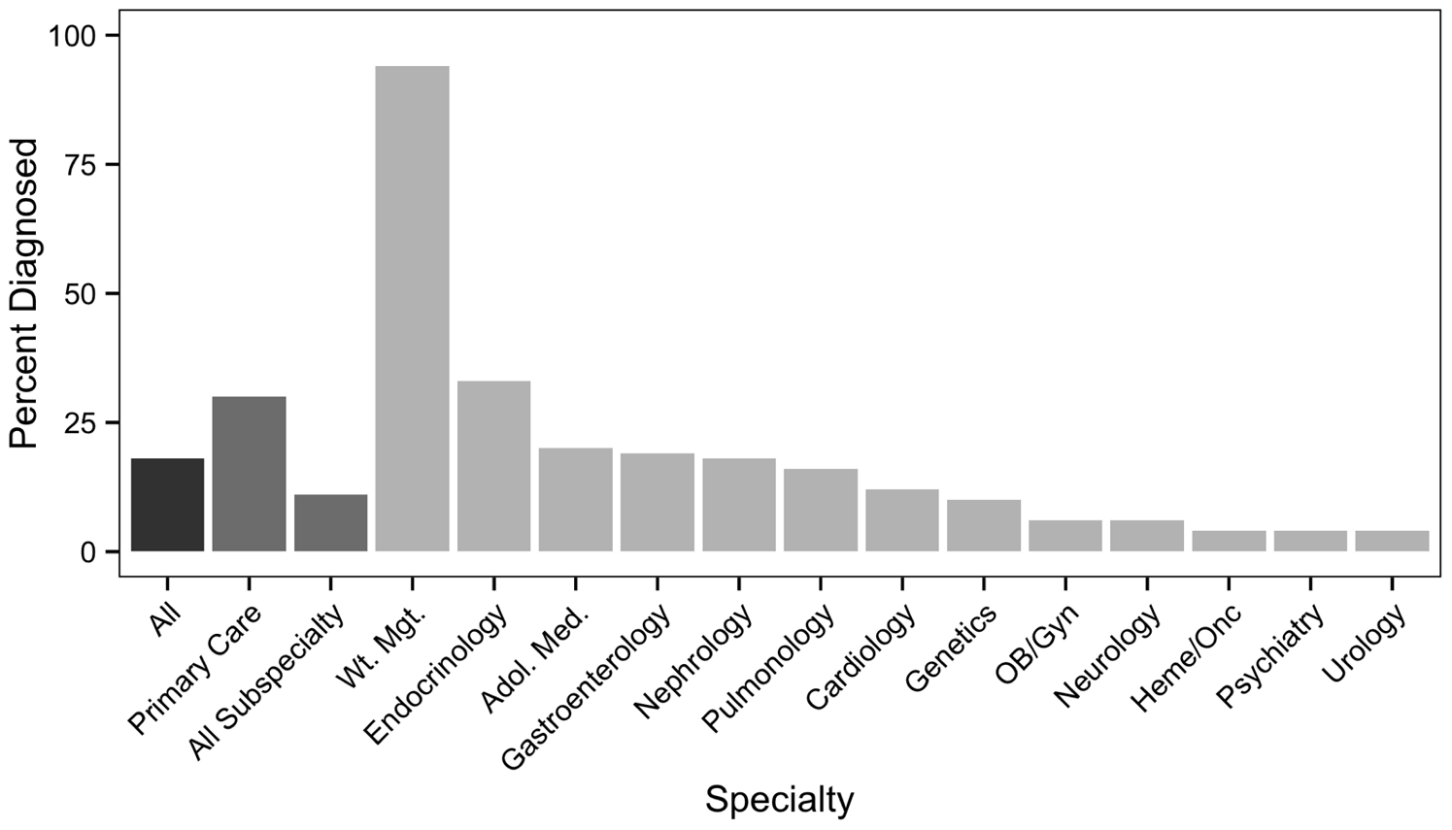
Diagnosis of Obesity at Outpatient Visits. Percentages of children who were obese at any time during the study period, and diagnosed as obese at any visit to the indicated specialty. All specialties with a diagnosis rate ≥4% are included.

**Figure 3** presents rates at which clinicians in different specialties made a diagnosis of obesity for children with elevated BMI. Overall, only 20% of children with one or more BMI measurements above the 95^th^ percentile had a diagnosis recorded at any visit. When the analysis was restricted to primary care visits, the rate rose to just 29%; considering only well child checks did not alter this result. The only contexts in which diagnosis rates exceeded 30% were endocrinology and weight management clinics. At the visit level, just 14% of all visits with measured obesity had a diagnosis of obesity recorded.

**Figure 4:**
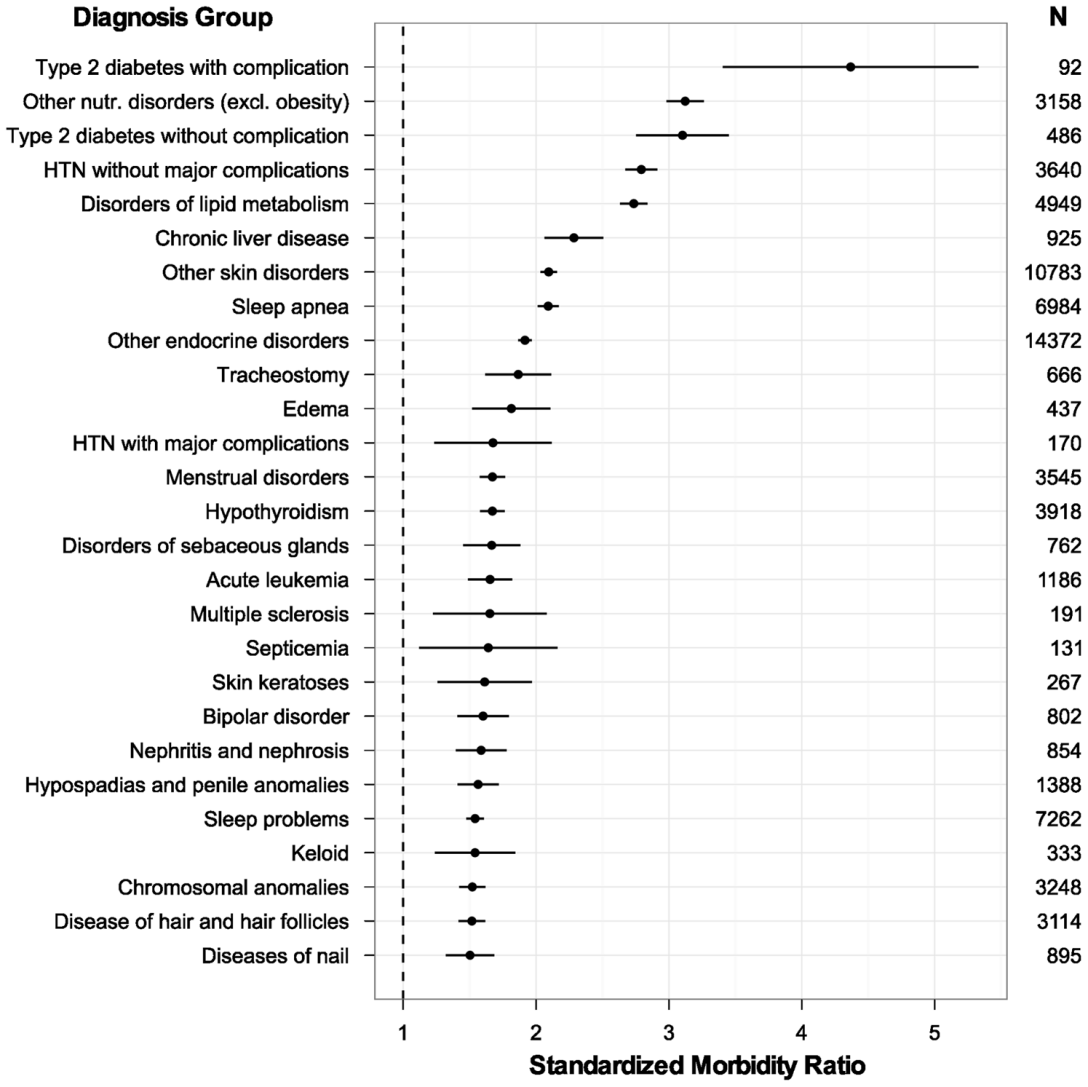
Obesity-Related Co-Morbidities. Standardized morbidity ratios (observed prevalence in obese children/expected prevalence from entire cohort) with 95% confidence intervals for diagnostic groups (EDCs) having SMR >1.5 and CI_95_>1.0 among children with measured obesity. **N** = total number of children in cohort with diagnosis in that EDC.

We used the EHR dataset to detect groups of conditions that most commonly co-occur with obesity (**Figure 4**). Several of these conditions are known comorbidities of obesity, such as hypertension and hyperlipidemia. However, we were also able to detect associations between obesity and rare disorders such as acute leukemia, multiple sclerosis, and chromosomal anomalies.

In addition, we observed an overall increase for obese children in both primary care visits (ever obese: 4.8±4.0 *vs.* never obese: 4.0±3.4; p<0.001) and specialty visits (3.7±6.6 *vs.* 2.7±4.0; p<0.001). After adjustment for age, sex, and site, 52% of this difference in outpatient utilization was attributable to diagnosed comorbidities, as assessed by ACG Resource Utilization Bands.

## Discussion

This study demonstrates the feasibility and validity of sharing EHR-derived data for assessing obesity in large populations of children. The effort required to retrieve the data was nominal, and largely for query development and validation, a one-time cost that would not apply to refreshing data for ongoing surveillance. The scale of EHR-derived data is significant: this sample from six pediatric centers produced 6,000 BMI assessments per month of age for most of childhood.

Aggregation of data across sites and EHR types represents an important test of principle. This study was designed with the intent to isolate technical and procedural factors that might affect the feasibility of data exchange. To that end, we focused on data types, such as anthropometric measurements, where the meaning of a value, as distinct from the method used to obtain it, was unambiguous. For clinical diagnoses, we used the International Classification of Disease, 9^th^ Edition, Clinical Modification (ICD9-CM) [36], the current standard diagnostic terminology in use in the United States. This provided a common vocabulary, though as noted below, usage of specific codes differed across institutions. In the general case, the problem of semantic interoperability [37], or accounting for the ways in which a common concept is represented in different contexts, remains a barrier to sharing of clinical information. Addressing this problem will require additional work in a number of areas, including development of more robust terminologies and standards for data interchange [37] [38], better understanding of ways in which clinicians interact with the EHR [39] and data are captured [40], and further studies of the operating characteristics of EHR-derived data.

Because most data in the EHR are obtained as part of routine clinical care, their structure will also reflect practice patterns that must be accounted for in secondary analyses. In our case, consistent with expected outpatient practice, patient heights were measured less often than patient weights. Where no height or weight data were available for a child, we considered them inevaluable for obesity, as it would be error-prone, and risk circularity, to impute these directly from a population distribution to an individual. However, unlike weight, for a given individual height velocity is a relatively stable physiologic quantity over the interval considered in this study. Therefore, when at least two measured heights were available, we used a conservative imputation strategy to derive height, and hence BMI, values for visits where it was not directly measured. Although this added no subjects to the dataset, it increased by 21% the number of evaluable visits available for longitudinal and practice type analyses with low risk to validity of data. It also reflects an anticipated, if not yet widely realized, benefit of EHR adoption: information from one site becomes more widely available for use at other sites sharing the EHR.

Secondary use of clinical data for research has also generated concern about the potential consequences of increased variability across measurements. Although the carefully controlled NHANES methodology likely does produce more precise individual measurements, the survey yields an average sample of 16 subjects per month of age. In the EHR data, the potential effects of individual measurement error are damped by the size of the sample and repeated measurement, resulting in highly stable population estimates of BMI and obesity prevalence compatible with NHANES results. This difference was particularly apparent in adolescents, where the EHR-derived data did not display the increased variance seen in NHANES measurements, which was likely due to the differences between individual children in timing of pubertal growth. Furthermore, at the individual level, we found high reliability for BMI assessments over time, suggesting that any error introduced by variation in assessment technique is small.

The cohort size achievable with EHR-derived data permits detection of clinically relevant associations not possible with survey data, such as the known association between acute lymphoblastic leukemia and secondary obesity [41]. EHRs also facilitate construction of cohorts with linked clinical data, to assess the impact of obesity on children with rare primary disorders. In these ways, EHR-based population surveillance can provide an important complement to in-depth but resource-intensive surveys such as NHANES.

It is important to note potential limitations of our EHR-based study. First, data are derived from six centers, leaving gaps in national geographic coverage and underrepresentation of rural areas. In particular, this may contribute to the difference from NHANES in prevalence of obesity in younger children; the higher proportion of measurements from children aged 2–10 in the EHR-derived data than in the NHANES sample is also in keeping with expected patterns of clinical utilization. In most respects, however, our estimates closely match the results of the NHANES stratified sampling model. A fortuitous combination of contributing sites is possible, though participating centers were not selected based on obesity prevalence. The means of 2.6 evaluable visits/child and 1.9 diagnoses/visit also indicate that our results were not likely to have been heavily biased by a subpopulation of children with complex medical conditions affecting their growth. Moreover, the low cost of EHR queries suggests that as the nation’s healthcare system becomes increasingly digitized, it will become possible to readily combine data from additional geographic areas and clinical settings, and increase the generalizability of results based on data sharing.

Second, the high data quality observed in the EHR dataset may in part reflect the selection of anthropometric and demographic data, which are semantically unambiguous and directly measured as a matter of routine in pediatrics, as the source of the primary outcome measured. Our results do provide significant reassurance against the concern that clinical data are generally too unreliable for use in research. However, the quality of other types of information, particularly subjective findings or clinical decisions, will depend on different sets of semantic and pragmatic considerations. Further study will be required to assess the fitness of these and other types of EHR-derived data for population-level analyses [42] [43].

Third, rates of obesity diagnosis in the EHR were remarkably low, even in primary care settings. Although unsurprising [44] [45] [46], this is a significant problem, since growth monitoring is a core function of pediatrics. We considered the possibility that our obesity-related ICD9-CM diagnosis cluster did not sufficiently comprise codes in common use. Examination of the most common diagnoses for obese children does not indicate that an alternative code(s), including those for overweight or specific BMI ranges, was used frequently (data not shown). Of note, the inclusion of 783.1 (“abnormal weight gain”) in our cluster is the result of this analysis demonstrating that it was the most common weight-related diagnosis given to obese children at one site. It is also possible that obesity is missing because multiple other diagnoses are recorded. However, obese children had on average 1.9 diagnoses/visit, a number unlikely to preclude adding a diagnosis of obesity. The low rates of diagnosis in primary care and well-child visits also argue that addressing more acute problems is not a major factor preventing diagnosis of obesity. We did observe stronger association between a diagnosis of obesity and many comorbid diagnoses than between measured obesity itself and these diagnoses (data not shown), suggesting that the presence of a comorbid condition such as hypertension or diabetes may “prompt” a diagnosis of obesity. Adding entries from the EHR’s problem list at the largest site increased the documentation rate by just 3%, showing that this is not a major alternative method of recording recognition of obesity. It is more likely that pediatricians are not recording obesity diagnoses for reasons other than lack of opportunity, such as a belief that obesity is best addressed by “non-medical” interventions, non-reimbursement of obesity diagnoses, or concern for stigmatization of patients. If we are to improve the quality of care for obese children, we will need to better document the problem in the medical record, where it serves not only as a cognitive marker, but as a trigger for additional decision support around appropriate screening and treatment.

EHRs provide access to primary clinical data, rather than specifically coded or prompted responses as on a case report form. This can bias ascertainment of a datum if it does not reflect a common element of clinical care. However, it can also be valuable, if it permits ascertainment of affected status directly, rather than relying on diagnoses or similar administrative data. As we demonstrate, using administrative data to identify obese children misses over 75% of affected individuals. It is possible that similar biases affect diagnoses of comorbid conditions used in our analyses. The strong associations seen between obesity and several known comorbidities are reassuring in this respect. However, further analyses using EHR-derived data can provide opportunities for direct assessment of other conditions, to more reliably establish association with obesity, and potentially to allow us to better identify subsets of children at higher risk for specific complications of obesity.

Using EHR data to monitor other aspects of population health will benefit increasingly from structured data in the EHR, such as diagnoses, vital signs, medications, and diagnostic results. Free text (*e.g.* clinical assessments and instructions) will require greater, though not necessarily prohibitive [21], effort to derive useful population-level information. In addition to data type, it will be important to understand operating characteristics of EHR-derived data, since the potential for selection and reporting biases will be different from other survey methods.

Using clinical information from the EHR, we demonstrate robust associations between measured obesity and diagnosed comorbidities such as diabetes and other endocrinopathies [47] [48] [49], hypertension [50] [51], dyslipidemia [52] [4], liver disease [53], and sleep apnea [54]. Obese children had increased overall healthcare utilization as well, about half of which is explained by excess diagnosed comorbidities. Both findings highlight public health implications of the high prevalence of obesity for children today and adults tomorrow. Further study to identify appropriate markers in the medical record of screening for and treatment of obesity-associated morbidity will help to define strategies for addressing these problems.

This study also suggests several opportunities for quality improvement. Overall, 44% of visits did not include sufficient data to assess BMI and 24% of subjects had no assessments over the two-year study period, which is recommended at least annually by the American Academy of Pediatrics as a universal practice [55], and is a core objective of the stage 1 meaningful use measures [56]. Only one in five obese children have the diagnosis recorded, an important step in the medical management of any condition. Moreover, the low opportunity cost of EHR-derived monitoring, potentially coupled with geocoding or other public health data, makes it possible to assess the impact of medical and community-based interventions on obesity in a variety of geographic and demographic settings. Methods validated using EHR-derived data can also provide direct input into design of decision support systems to improve quality at the point of care.

### Conclusion

We are still early in the process of incorporating the EHR into clinical and public health practice. This study demonstrates the potential for integrating EHR-derived data from multiple sources to monitor childhood obesity and its correlates. Given the breadth of information collected in EHRs, we believe this potential extends to many areas of population health management; utility for specific conditions will depend on the degree to which critical data are consistently captured and can be meaningfully recovered from the EHR. Further, the close linkage of source data to patient care may allow systems that incorporate EHR-derived data to more effectively translate results into clinical practice. Developing a nationwide cross-institutional data sharing system holds the potential for population health surveillance, quality improvement, and ultimately formation of the digital infrastructure of a transformative, learning health system for the nation [5]. Both health information exchanges and clinical research networks such as HMORN and PEDSNet will contribute to understanding the logistical and scientific requirements for effective use of clinical data in this process.
